# Gaps in Developmental Pediatrics training: A Canadian resident physician perspective

**DOI:** 10.15694/mep.2018.0000136.1

**Published:** 2018-06-21

**Authors:** Maria Cristina Tassone, Thivia Jegathesan, Ra Han, Adelle Atkinson, Stella Ng, Elizabeth Young

**Affiliations:** 1 Department of Pediatrics; 2 Institute of Medical Sciences; 3 Department of Pediatrics; 4 Faculty of Medicine; 5 Department of Pediatrics

**Keywords:** developmental pediatrics, competency-based medical education, postgraduate medical education, child development

## Abstract

This article was migrated. The article was marked as recommended.

**Introduction:** Postgraduate medical training worldwide has recently experienced a transition to Competency-Based Medical Education (CBME). This provides a timely opportunity to critically evaluate the postgraduate medical curriculum, particularly from a trainee perspective. Studies reveal that Canadian residents and recent graduates in pediatrics and family medicine are uncomfortable with their proficiency in child development. However, little is known about residents’ perceptions of their training, nor where specific needs lie. We therefore sought to identify gaps in developmental pediatrics training, with the goal of informing the development of a new CBME curriculum.

**Methods:** An online cross sectional needs assessment survey was administered to current pediatrics and family medicine residents at our institution. A total of 63 residents participated, 43 pediatrics and 20 family medicine.

**Results:** Four key themes emerged from analysis of survey results: 1. Residents agree that developmental pediatrics is relevant to future practice and competency; 2. Residents feel they lack competency in the assessment and management of patients with developmental issues; 3. Residents’ feelings of insufficient and inadequate training increase over time; 4. Residents recommend changes to developmental pediatrics training.

**Conclusion:** As we prepare to transition to CBME, curriculum should be purposefully developed to meet resident identified need and reflect appropriate competencies required for clinical practice.

## Introduction

In recent years, postgraduate medical training worldwide has experienced a shift from the traditional time-based model of knowledge and skills acquisition, to Competency-Based Medical Education (CBME) ( Caraccio et al 2004; Ten Cate and Scheele 2007; RCPSC 2014). CBME shifts the focus to outcomes-based training, with an emphasis on progressively advancing skills and regular assessment of performance within a timeframe that is more individualized to each trainee ( RCPSC 2014). Both the Royal College of Physicians and Surgeons of Canada and the College of Family Physicians of Canada are currently in the process of implementing this model ( CFPC 2011; RCPSC 2017). This transition provides a timely opportunity to critically evaluate the postgraduate medical curriculum and assess residents’ opinions on their education at our institution. Among many postgraduate programs, our institution trains family medicine and pediatric residents within a large, urban, multicultural Canadian center. Both disciplines include a focus on child development and developmental disorders across the lifespan. We use this key area of pediatric medicine as an example of how the opinions of trainees can, and should, be used to inform CBME curriculum development.

As front line physicians, family doctors in Canada are responsible for monitoring children’s development and recognizing red flags that warrant a developmental assessment. In most areas of Canada, pediatricians diagnose common developmental issues and provide recommendations for management. Family physicians provide long-term follow-up, access to therapies and resources, and ongoing monitoring. Pediatrics residents at our institution have a 4-week block dedicated to Developmental Pediatrics in their second year of training. During this time, they participate in a variety of outpatient clinics at a tertiary pediatric center for development and rehabilitation. They have several other ad hoc and elective opportunities in their senior years, and receive 1-2 lectures per year on Developmental Pediatrics. Family medicine residents have a 4-week pediatric rotation in their first year of training. During this rotation, they are expected to learn pediatrics as a whole, including development. They participate in 3-4 half-day child development clinics and may pursue additional electives. They receive 2-3 lectures per year on topics in pediatrics, including child development. This program format is similar across Canadian residency programs ( CFPC 2011; RCPSC 2015).

In the context of their current training, studies reveal that Canadian residents and recent graduates in pediatrics and family medicine report feeling uncomfortable with their proficiency in areas of child development and behaviour ( Gold and Shaw, 2003; Boreman et al 2007; Grant et al. 2007; Golnik et el 2009; Carbone et al. 2010; Wilkinson et al. 2012). Several studies attribute physician discomfort to deficiencies in postgraduate developmental pediatrics training ( Gold and Shaw, 2003; Boreman et al 2007; Grant et al. 2007; Golnik et el 2009). However, little is actually known about trainees’ perceptions of their training in child development, nor where specific gaps and needs lie ( Lieberman and Hilliard 2006; Grant et al. 2007; Carbone et al. 2010; Rosenberg et al. 2011; Wilkinson et al. 2012). We therefore sought to identify and define gaps in developmental pediatrics training perceived by both pediatrics and family medicine residents at our large urban Canadian center, with the goal of informing the development of a new competency-based curriculum for child development.

## Methods

### Design

An online cross sectional needs assessment survey was emailed to current pediatrics residents (PGY1 - PGY4) and family medicine residents (PGY1-PGY2) at our centre. Fourth year pediatrics residents included those in the general pediatrics stream and first year of subspecialty fellowship training, as they both write their pediatrics certification exam at the end of their fourth year. Reminder e-mails were sent out at one week and one month. Hard copies of the survey were also offered to residents at one of each program’s academic days.

### Survey

The survey addressed: 1) perceived importance of child development to physician knowledge and future practice, 2) self-perceived competence in the assessment and management of children with developmental concerns, 3) perceived sufficiency and adequacy of current training, and 4) desire for additional training. Likert scale responses and three open-ended questions allowing free text responses were used.

### Data Analysis

The results were analyzed by three study personnel (MT, TJ, EY) using thematic analysis to identify and define common themes in survey responses. Demographics and overall resident perceptions about their training across both family medicine and pediatric residents were analyzed using description statistics with SPSS V 21. This data was calculated using proportions and percent to identify overall patterns of perception among residents at our centre.

### Response Rates + Demographics

A total of 113 residents (72 pediatric and 41 family medicine) were invited to participate in the survey. Response rates were 55.8% overall (63/113), 59.7% among the pediatrics residents (43/72) and 48.8% (20/41) among the family medicine residents. Table 1 shows participant demographics.

**Table 1.** Participant demographics

	Pediatrics (n=43)	Family Medicine (n=20)
Sex (%)		
Male	15 (35)	5 (25)
Female	28 (65)	15 (75)
Postgraduate year of training (%)		
PGY1	12 (28)	10 (50)
PGY2	15 (35)	10 (50)
PGY3	7 (16)	-
PGY4 core pediatrics	5 (12)	-
PGY4 subspecialty pediatrics	4 (9)	

### Ethics

This study was approved by the Research Ethics Boards of Providence Healthcare, St. Joseph’s Health Centre and St. Michael’s Hospital, REB 15-060.

## Results

### Theme 1: Residents agree that developmental pediatrics is relevant to future practice and competency

The majority of both pediatrics (93.0%) and family medicine (80.0%) residents felt that knowledge of normal and abnormal child development was relevant to their future practice. All pediatrics (100%) and most family medicine residents (95.0%) felt that physicians in their field should be able to recognize red flags in child development. Both pediatrics (88.4%) and family medicine (70.0%) residents agreed that physicians in their field should be competent in managing uncomplicated cases of developmental diagnoses without referral to a developmental specialist.

*“Understanding developmental pediatrics really establishes a foundation for understanding pediatrics in general. We are going to be the physicians to assess these patients first in the community..More experiences in the community would be most beneficial, especially to learn skills and tools useful for general practice”, - PGY2 pediatrics resident*

### Theme 2: Residents feel they lack competency in the assessment and management of patients with developmental issues

Our survey results show that the areas of lowest self-perceived competency correspond directly to the predominant roles of pediatricians and family doctors in clinical practice. Only 22.2% of pediatric residents in their final year of training reported feeling competent in assessing patients with a developmental concern. None (0%) of the family medicine residents in their final year of training reported feeling competent in long term management of patients after a diagnosis is made, or discussing support services with families.

**Figure 1.**
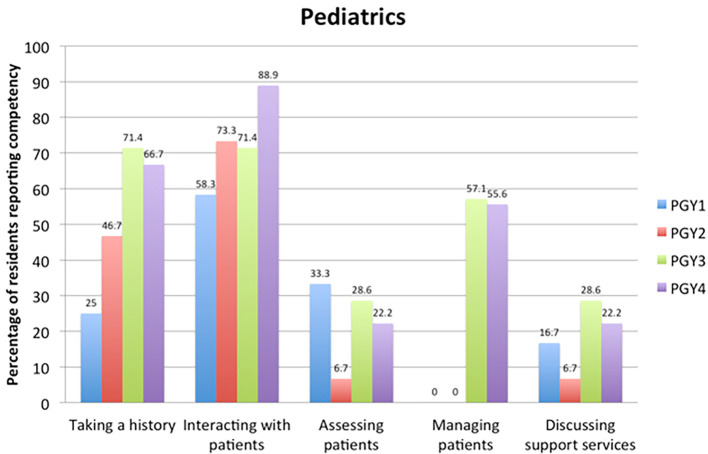

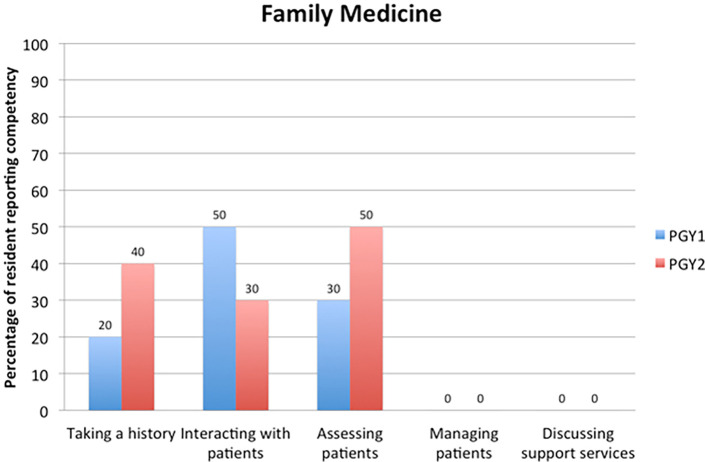
Self-perceived competency in areas of developmental pediatrics by postgraduate year among pediatrics ( Figure 1a) and family medicine ( Figure 1b) residents

Most fourth year pediatrics residents reported feeling competent in taking a history (66.7%) and interacting with patients (88.9%) with a developmental concern. However, among second year family medicine residents, only 40% reported feeling competent in taking a history and 30% in interacting with patients. ( Figure1)

*“I feel the part that’s lacking is how to manage and where to refer these families for support. I do not feel well prepared in terms of connecting these families with the support they need”. -PGY2 family medicine resident*

### Theme 3: Residents’ feelings of insufficient and inadequate training increase over time

**Figure 2.**
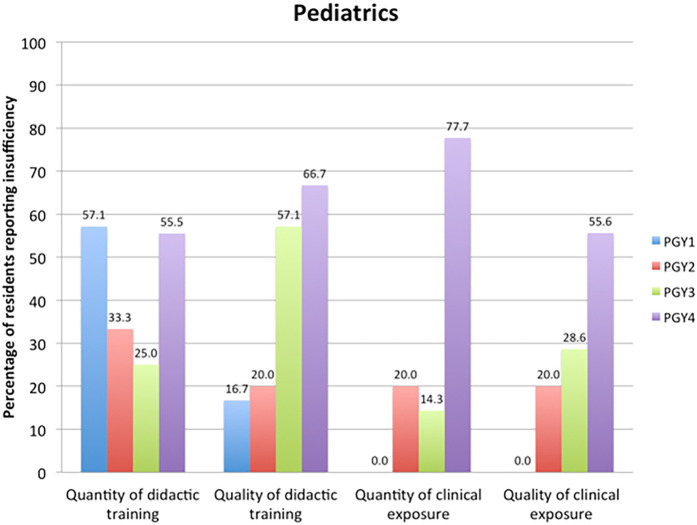

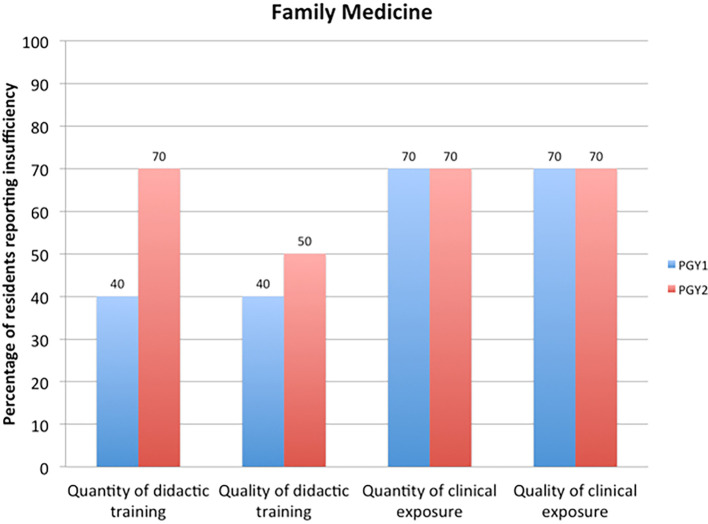
Perceived insufficiency of quantity and quality of didactic training and clinical exposure in developmental pediatrics by postgraduate year among pediatrics ( Figure 2a) and family medicine ( Figure 2b) residents

Most residents reported that the quality and quantity of both didactic training and clinical exposure in developmental pediatrics is insufficient. When assessed by year of residency, this feeling did not improve as training progressed. In fact, reports of insufficient training increased by year of Pediatric training. Among family medicine residents, reported insufficiency either increased or remained the same in the second and final year of training. ( Figure 2)

**Figure 3.**
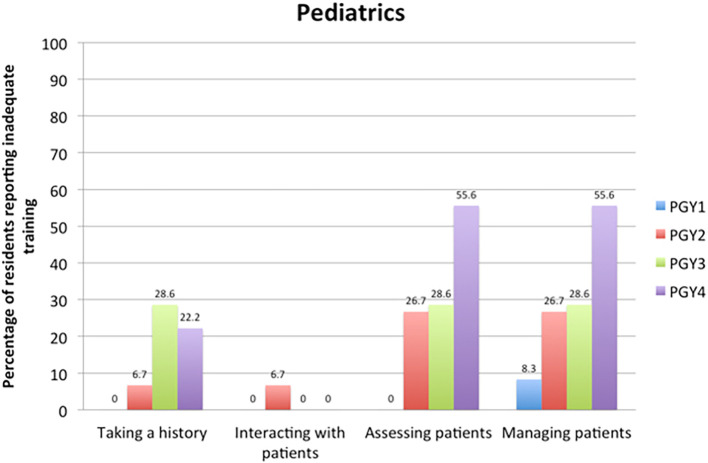

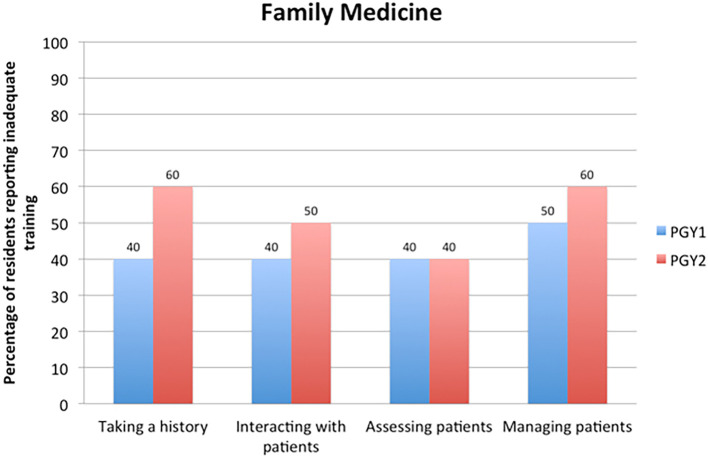
Perceived inadequacy of training in developmental pediatrics by postgraduate year among pediatrics ( Figure 3b) and family medicine ( Figure 3b) residents

Most residents reported that training in each skill area of developmental pediatrics is inadequate. Again, this feeling did not improve among pediatrics residents who were more advanced in their training. In areas of patient assessment and management, reports of inadequate training increased with each year of residency. ( Figure 3)

*“The number of opportunities that we have to perform primary focused developmental assessments is significantly lacking”. - PGY3 pediatrics resident*

### Theme 4: Residents recommend changes to developmental pediatrics training

Most residents (93% pediatric, 100% family medicine) agreed they would benefit from additional training in developmental pediatrics. Residents offered several suggestions to improve training including: early introduction to developmental pediatrics, increased clinical exposure in a greater variety of settings, and longitudinal training throughout residency.

*“I think having developmental pediatrics in first year would establish foundations for understanding pediatrics in general. More community developmental pediatrics would be beneficial to provide a breadth of clinical experience”. - PGY2 pediatrics resident*

*“I really think developmental pediatrics training should be throughout residency and not necessarily lumped into a specific year”. - PGY1 pediatrics resident*

## Discussion

This is one of the first studies in Canada to investigate trainees’ perceptions of child development training at a large urban center, and demonstrates the potential benefit of CBME in this area of pediatrics.

Although trainees recognize the significance of developmental pediatrics in their fields, pediatrics and family medicine residents at our center do not feel competent in key areas required for future practice. It is especially concerning that areas of greatest discomfort correlate with those most relevant to the role of a consultant pediatrician (diagnosis) and family physician (long term follow up) in the care of children with developmental concerns. Our residents reported that their training in child development is both insufficient in its overall quantity and quality, and inadequate in specific skill areas. Furthermore, these feelings of discomfort increase with progression through residency, which may be reflective of a greater awareness of skills that will be required for practice. While prior studies infer that this discomfort is a result of deficiencies in training, our survey demonstrates a clear link between feelings of incompetence and dissatisfaction with current training.

Issues in development and behaviour are among the most prevalent health concerns in children worldwide and account for a significant proportion of visits to family physicians and pediatricians ( World Health Organization 2013). This underscores the importance of having these physicians feel competent in managing children with developmental concerns. Discomfort among front line physicians may contribute to larger volume of referrals to developmental specialists, while wait times to accessing developmental subspecialty assessment is being increasingly recognized as an issue. Studies in the United States have found average wait times of 3-6 months (Bisgaier et al. 2011; Jimenez et al. 2017), and in Canada wait times have been reported up to 12 months ( Office of the Auditor General of Ontario 2013). While reasons for delays in access are multi-factorial, some of the burden may be mitigated by enabling family physicians and pediatricians to manage most cases, and only referring those with higher complexity to subspecialists. Similar to mental health, development must be enhanced within primary care practice if more families are to be provided timely care.

There is a clear need for improvement in postgraduate developmental pediatrics training in order to address these issues, and CBME is an ideal means to this end. CBME curriculum consists of a several “milestones”, or skills and abilities that residents should possess at each stage of training along of the spectrum of novice to expert ( RCPSC 2014). Key tasks called “entrustable professional activities” encompass a number of milestones and are observed by supervisors to evaluate competence ( RCPSC 2014). The breadth and complexity of developmental pediatrics requires that specific skills be taught and practiced over time in order to be incorporated into practice. Milestones can be developed that correspond to specific skills required for practice, with a particular focus on those identified by residents as currently being inadequately taught. These milestones should be individualized to each residency program based on scope of future practice. For example, Family Medicine training could focus on the long term management and supports for these families, whereas the Pediatric training could focus on diagnostic assessment. Our study shows that as residents become more aware of their roles, they also have increased feelings of inadequacy of training in these areas. By requiring progressively advancing milestones to be met in specific areas as training proceeds, competency will be built over time and resident satisfaction may therefore improve.

In our study, specific suggestions were made by residents for improvements in training. These include early introduction to developmental pediatrics, increased clinical exposure in a variety of settings, and focused longitudinal training including long term follow up for general practitioners and diagnostics for pediatricians. These changes would be easily incorporated into a CBME curriculum by developing skill-specific milestones in developmental pediatrics that begin early in training and continue throughout, requiring residents to have ongoing exposure, practice and evaluation of these skills.

Although our study only reflects developmental pediatrics training in the Canadian context, issues in child development and behaviour and the ongoing need for high quality, timely care for these patients is relevant worldwide. Internationally, we are beginning the process of unifying medical education with the goal of modernizing and enhancing physician training. Curriculum changes will undoubtedly need to reflect local practice needs, however a central commonality exists. In order to make the most success of CBME, obtaining resident opinion is a crucial first step to identify gaps in training and competency, and develop new curriculum to meet these needs.

## Conclusions

Our study identifies resident opinion on current Developmental Pediatrics training and how a CBME model of training can be used to address identified gaps. Current postgraduate training in Developmental Pediatrics at our institution leaves residents feeling uncomfortable and incompetent in their clinical skills, particularly in areas specific to future practice. Perceptions of inadequate of training increases as they progress through their residency programs. As we prepare to transition to CBME, curriculum should be purposefully developed to meet resident identified need and reflect appropriate competencies required for clinical practice. Like the specialty of Developmental Pediatrics itself, CBME curriculum can be tailored to reflect the steady progression and achievement of milestones for the trainee, so that skills are increased and sustained over time.

## Notes On Contributors

Dr. Maria Tassone is a Pediatrics resident in her last year of training at the University of Toronto. Her reasearch has focused on improving care in developmental pediatrics through clinical and educational initiatives. After residency, she plans to practise general pediatrics with a special interest in child development and behaviour.

Thivia Jegathesan is a Phd Student at the University of Toronto studying neonatal jaundice in neonates at St.Michael’s Hospital. She is also a medical education fellow at Women’s College Hospital under the Centre for Ambulatory Care Education studying reflective practice in developmental pediatrics.

Dr. Ra Han is a pediatric cardiologist at St. Michael’s Hospital and the Hospital for Sick Children. She is also the director of Pediatric Medical Education at St. Michael’s Hospital.

Dr. Adelle Atkinson is an Associate Professor of Paediatrics at the University of Toronto and in the Department of Paediatrics. She is a clinician educator, currently responsible for and overseeing the core training for the General Paediatric Residents. Her interest in this particular project is around gaps in training that can be addressed by curriculum reform.

Dr. Stella Ng is the Arrell Family Chair in Health Professions Teaching and the Director of Research, Centre for Faculty Development, St. Michael’s Hospital and University of Toronto. She is also a Scientist with the Wilson Centre and Assistant Professor, Speech-Language Pathology, University of Toronto.

Dr. Elizabeth Young is a general consulting and developmental pediatrician in the Department of Pediatrics at St.Michael’s Hospital. She is an Assistant Professor at the University of Toronto. Her work experience in both developmental and general pediatrics provides her a unique perspective in the management of families of children with developmental disorders.
