# Supplementary material for: Gaps in Developmental Pediatrics training: A Canadian resident physician perspective
Source: MedEdPublish (2016). 2018 Jun 21;7:136. [Version 1] doi: 10.15694/mep.2018.0000136.1 (PMC10701814; doi:10.15694/mep.2018.0000136.1)
Supplement: Peer review history

# Reviewer report: Reviewer response for version 1

**DOI:** 10.21956/mep.19581.r29062

**Published:** 2018-06-23

**Authors:** Peter Dieter

**Affiliations:** 1 AMSE

This review has been migrated. The reviewer awarded 4 stars out of 5 This study reports about gaps in developmental pediatrics training in the Canadian University of Toronto. Residents were asked to identify deficiencies in their current curriculum and should give examples. They agreed that developmental pediatrics is relevant to future practice and competency, they lack competency in the assessment and management of patients with developmental issues, they agreed on insuﬃcient and inadequate training increase over time and they recommend changes to developmental pediatrics training.The paper contributes thereby to the development of a CBME curriculum

Reviewer Expertise:

NA

No decision status is available

# Reviewer report: Reviewer response for version 1

**DOI:** 10.21956/mep.19581.r29061

**Published:** 2018-06-21

**Authors:** Ronald M Harden

**Affiliations:** 1 AMEE

This review has been migrated. The reviewer awarded 4 stars out of 5 Studies such as this one which have the potential of contributing to a competency based curriculum are of value. CBME should take account the views of the different stakeholders - in this case the residents. From the perspective of the residents important deficiencies in the current curriculum were found. This became more apparent as the training proceeded. While estimates of the residents confidence in the different areas is important it would have been useful to see how this correlated with objective measurements eg in an OSCE.. A follow up report would be interesting to see how the important results in this study are reflected in a competency based programme and in EPAs. There are some mistakes in the references - Wilkonson should be Wilkinson and Tripe C competency should be Triple C but this does not detract from the value of the study.

Reviewer Expertise:

NA

No decision status is available
